# Supplementary figures and images for: DENV-specific IgA contributes protective and non-pathologic function during antibody-dependent enhancement of DENV infection
Source: PLoS Pathog. 2023 Aug 28;19(8):e1011616. doi: 10.1371/journal.ppat.1011616 (PMC10491401; doi:10.1371/journal.ppat.1011616)

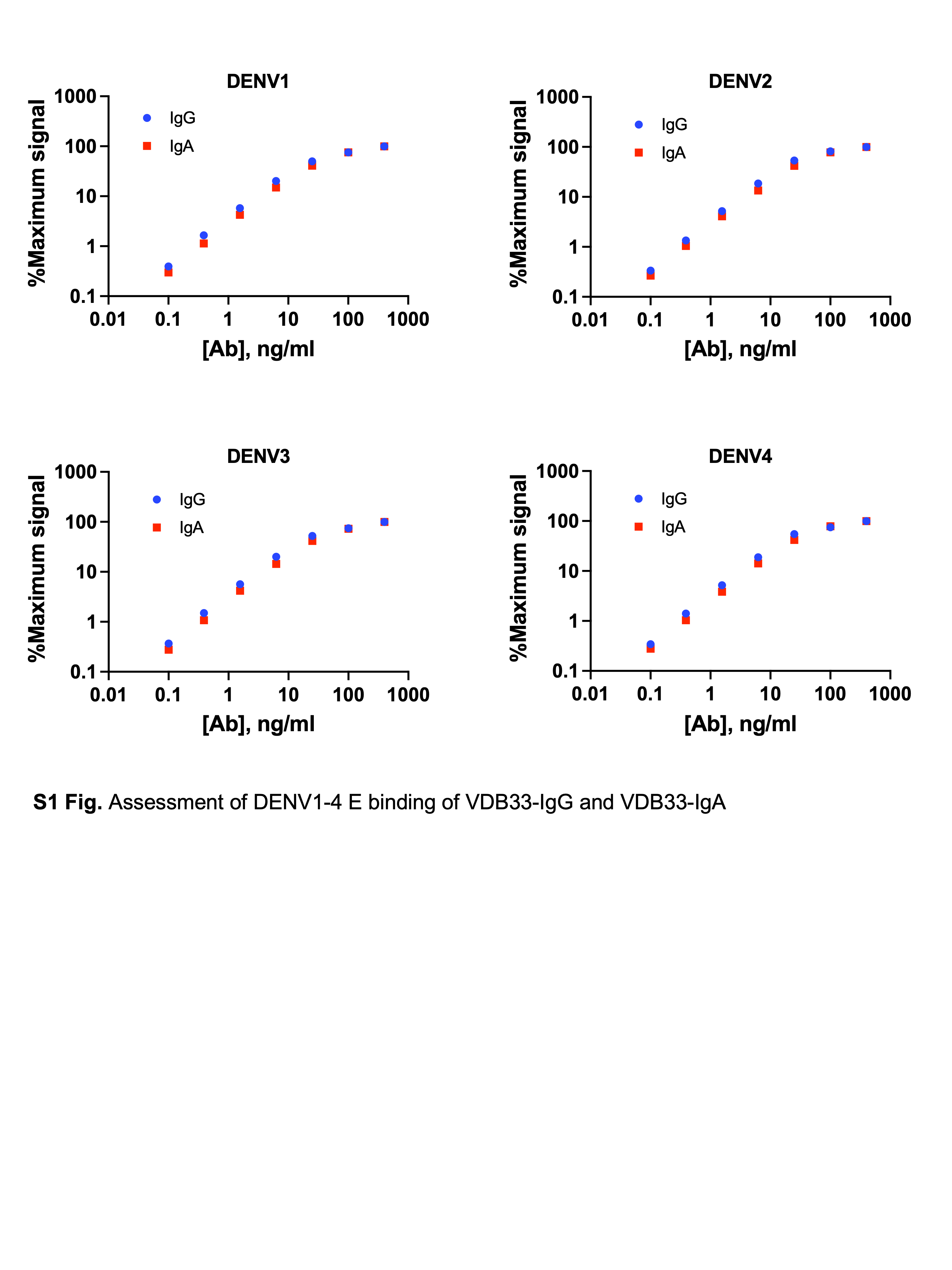

Supplement: S1 Fig — (TIFF) [file ppat.1011616.s001.tiff]

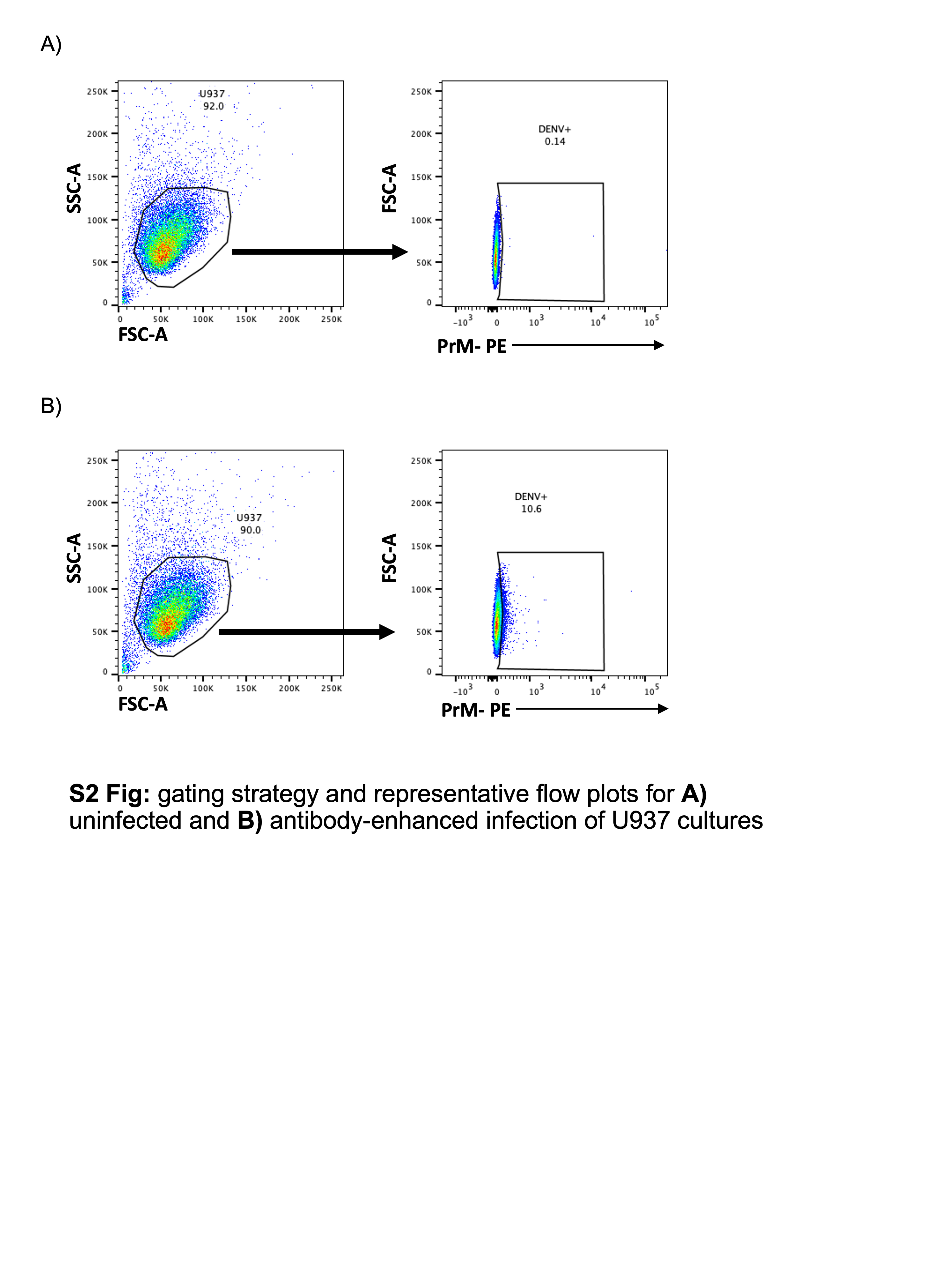

Supplement: S2 Fig — (TIFF) [file ppat.1011616.s002.tiff]

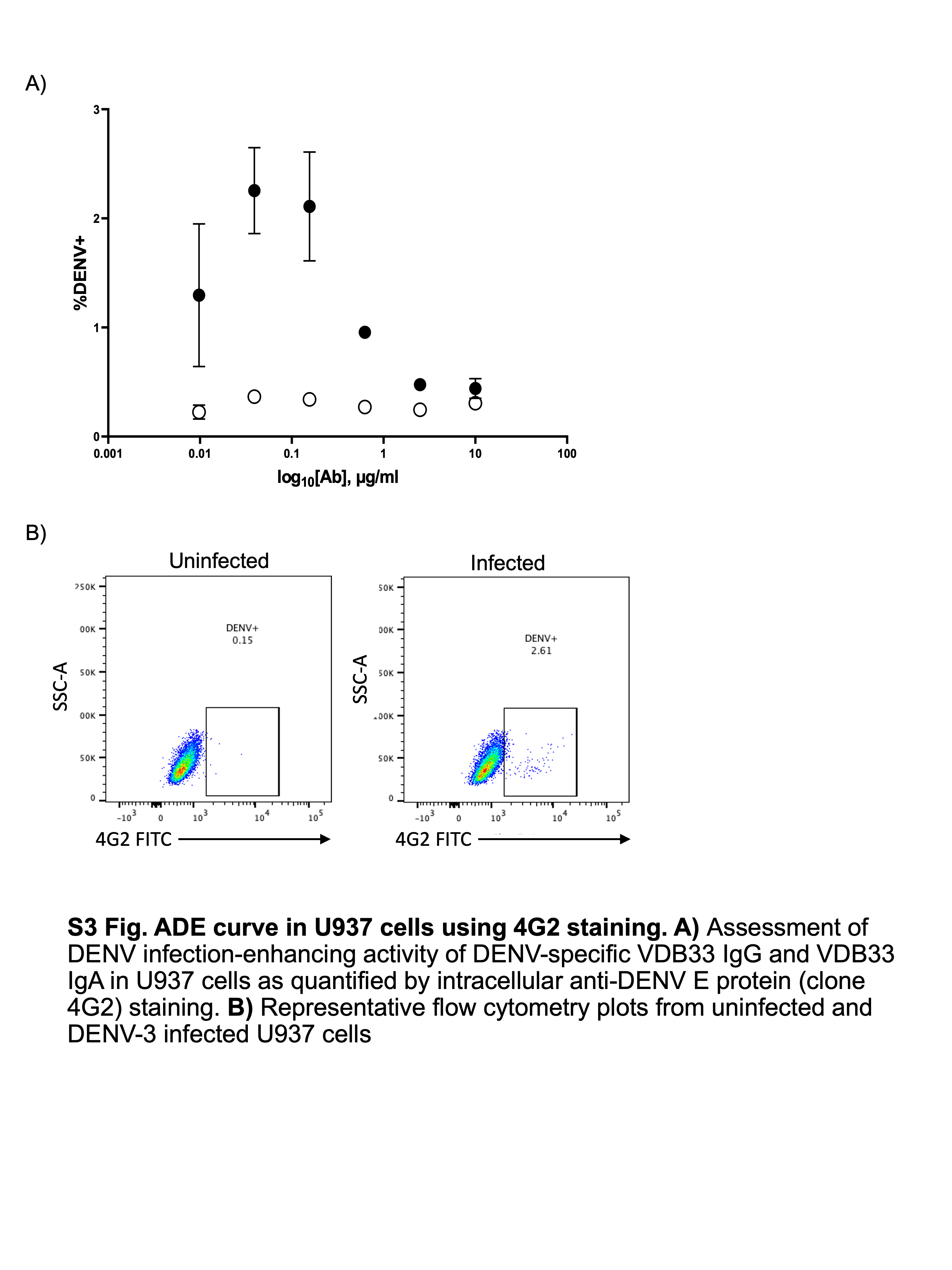

Supplement: S3 Fig — (TIFF) [file ppat.1011616.s003.tiff]

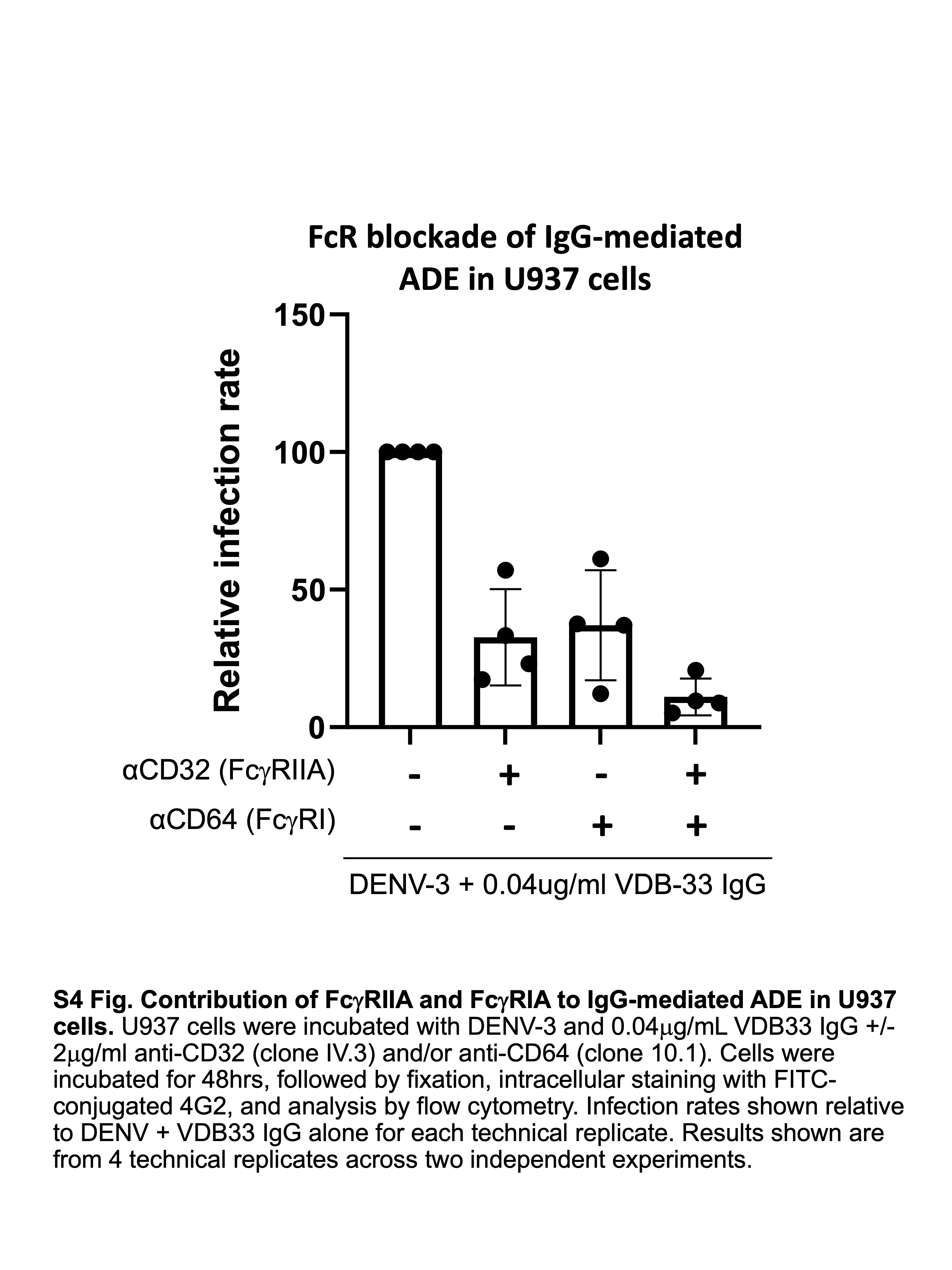

Supplement: S4 Fig — (TIFF) [file ppat.1011616.s004.tiff]

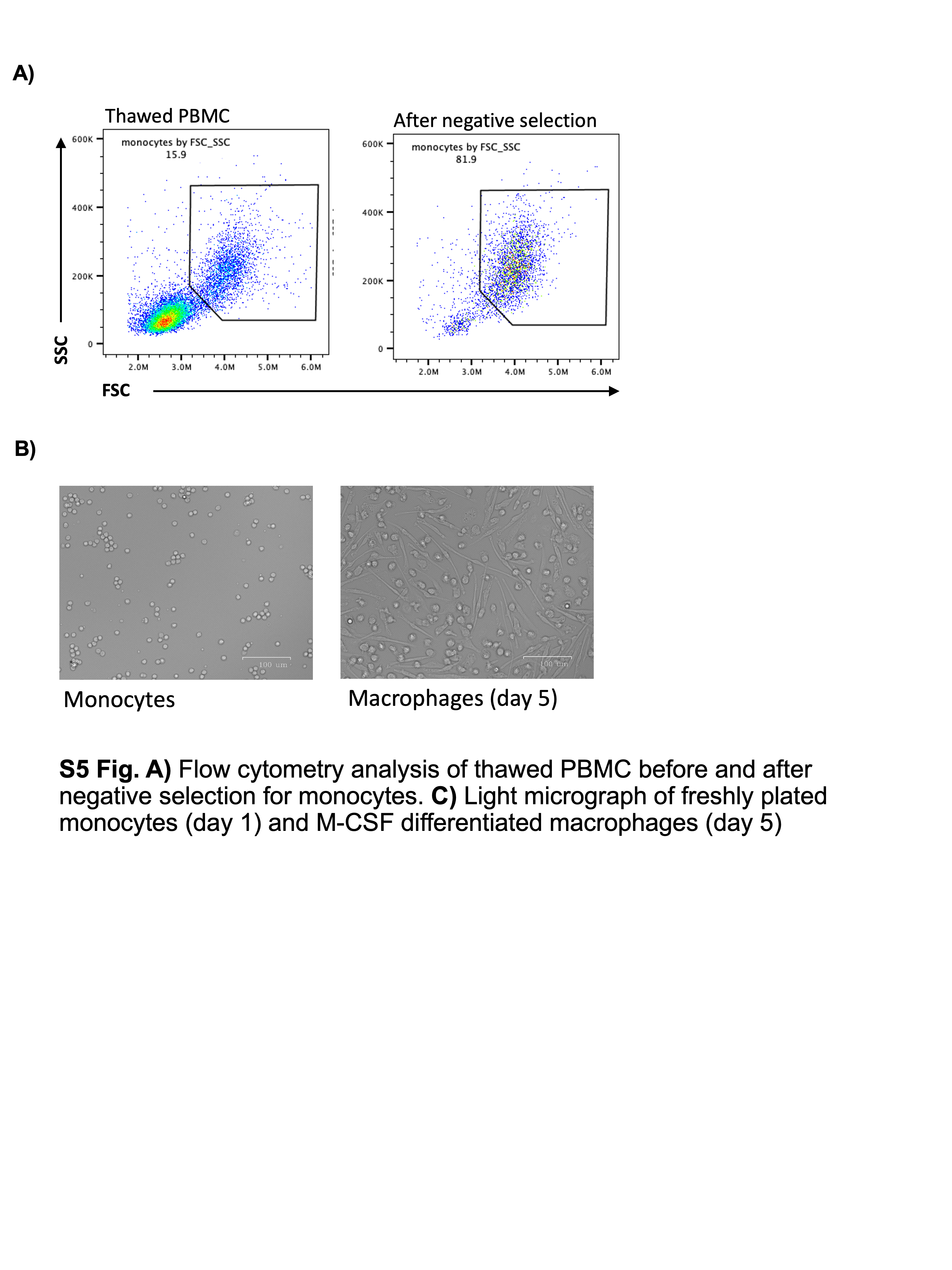

Supplement: S5 Fig — (TIFF) [file ppat.1011616.s005.tiff]

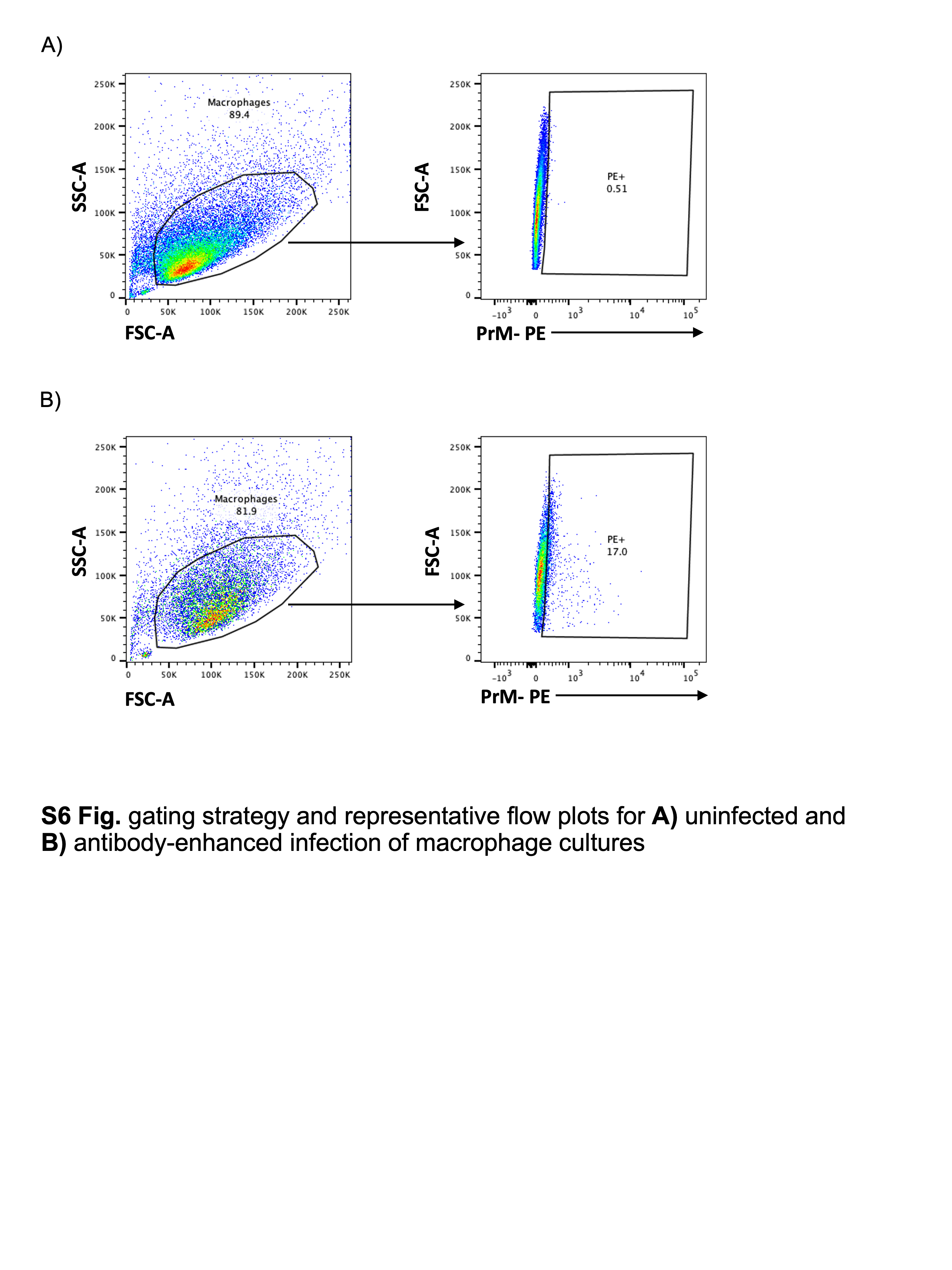

Supplement: S6 Fig — (TIFF) [file ppat.1011616.s006.tiff]

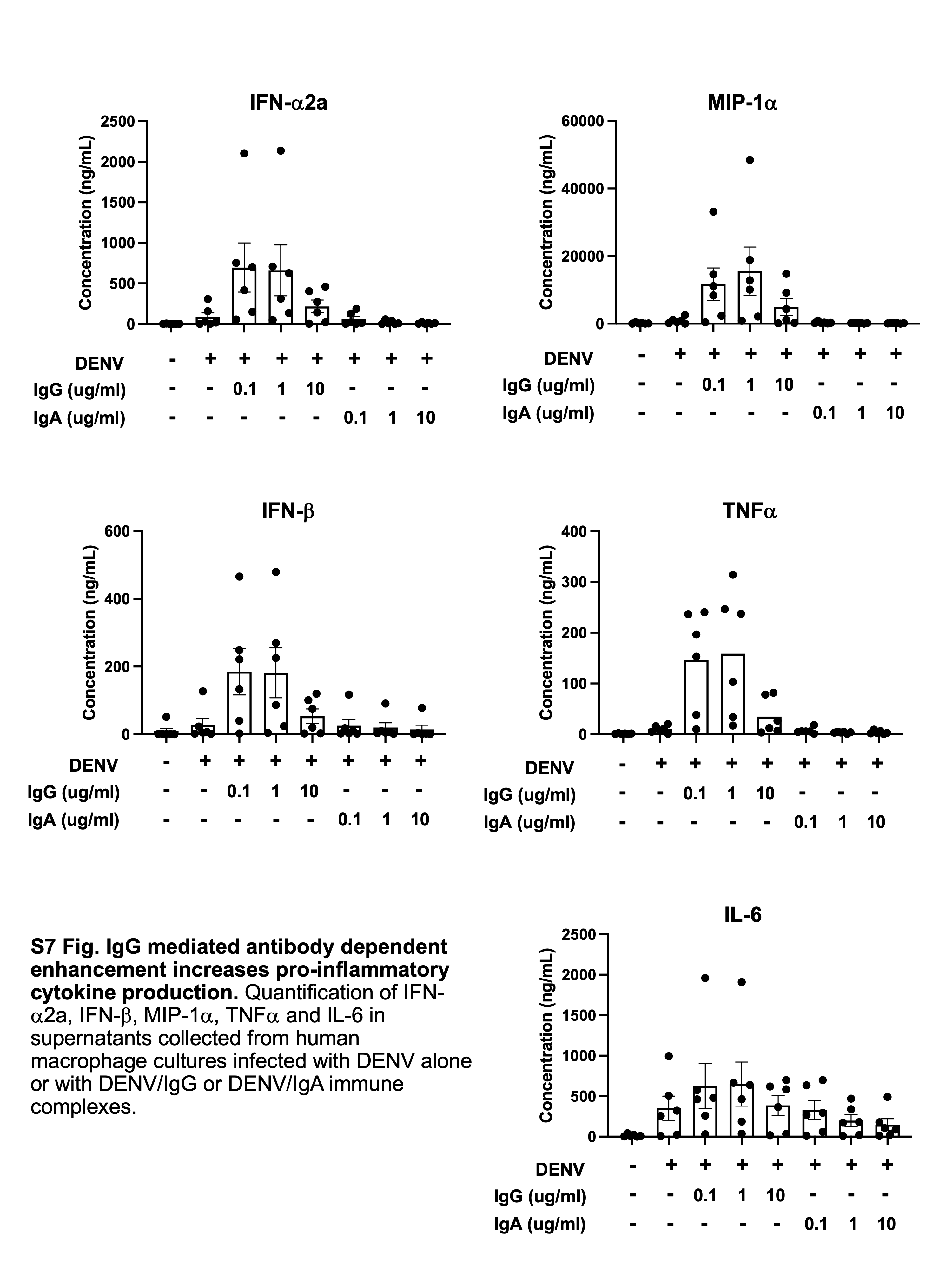

Supplement: S7 Fig — (TIFF) [file ppat.1011616.s007.tiff]

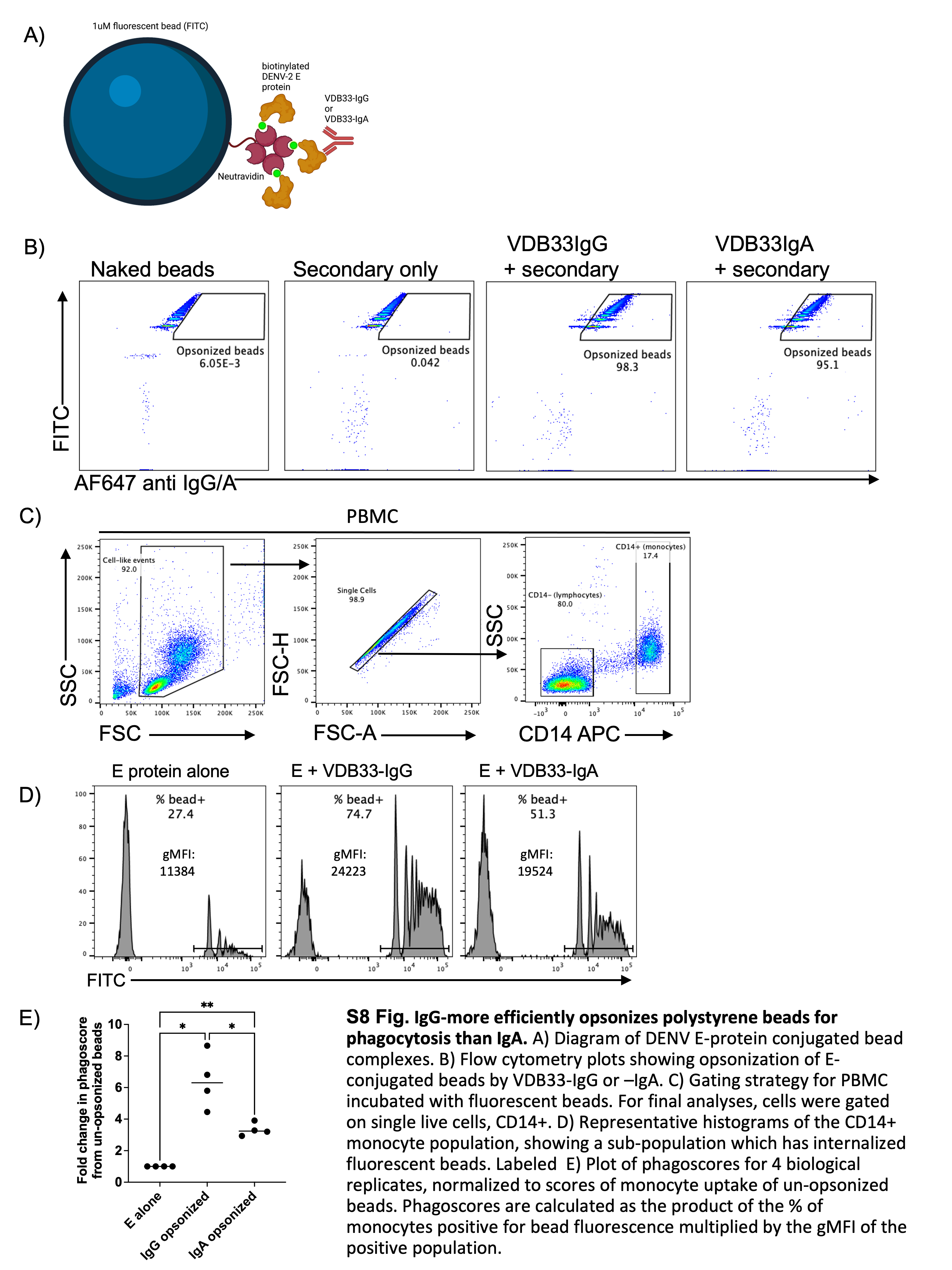

Supplement: S8 Fig — (TIFF) [file ppat.1011616.s008.tiff]

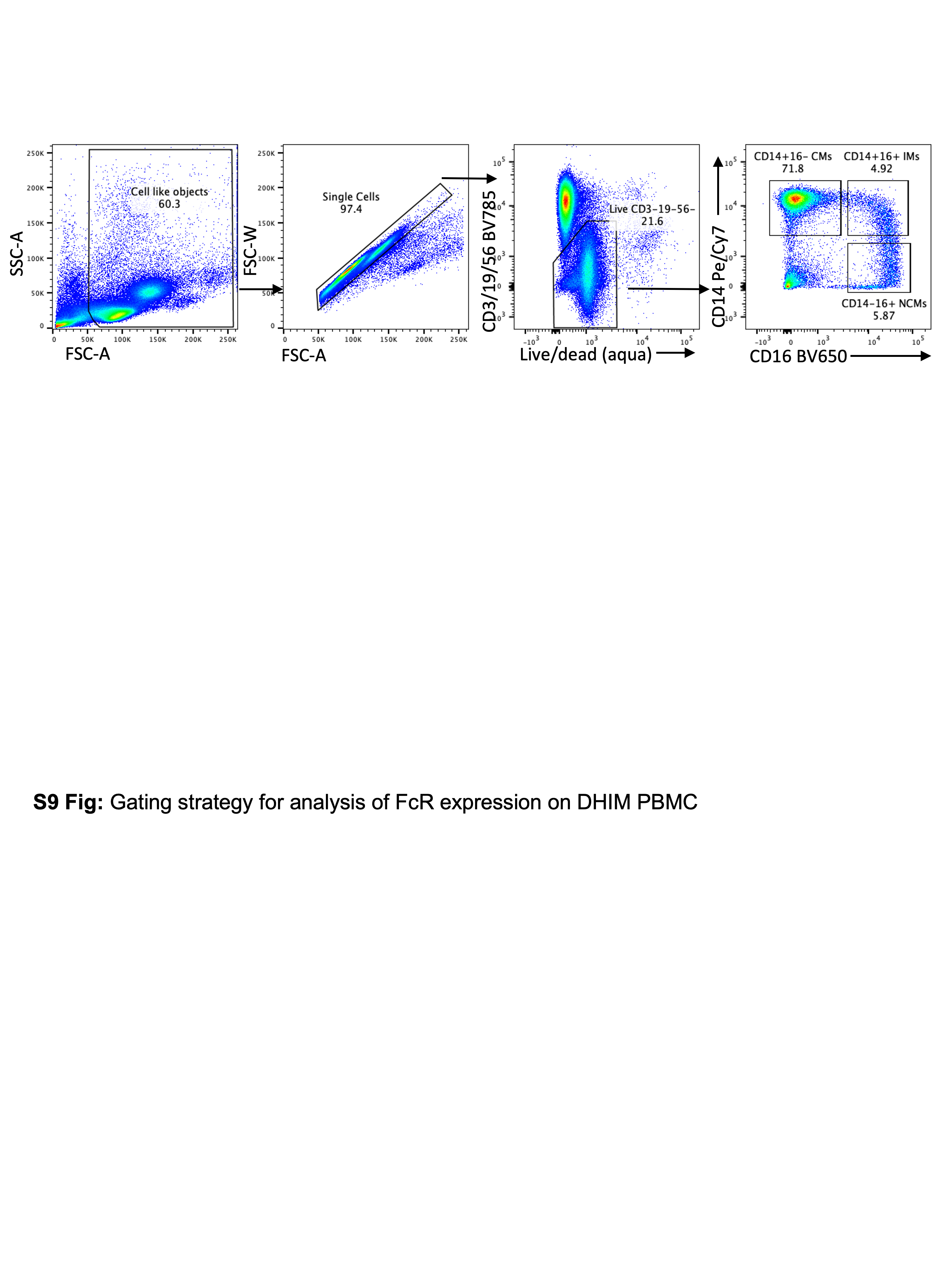

Supplement: S9 Fig — (TIFF) [file ppat.1011616.s009.tiff]

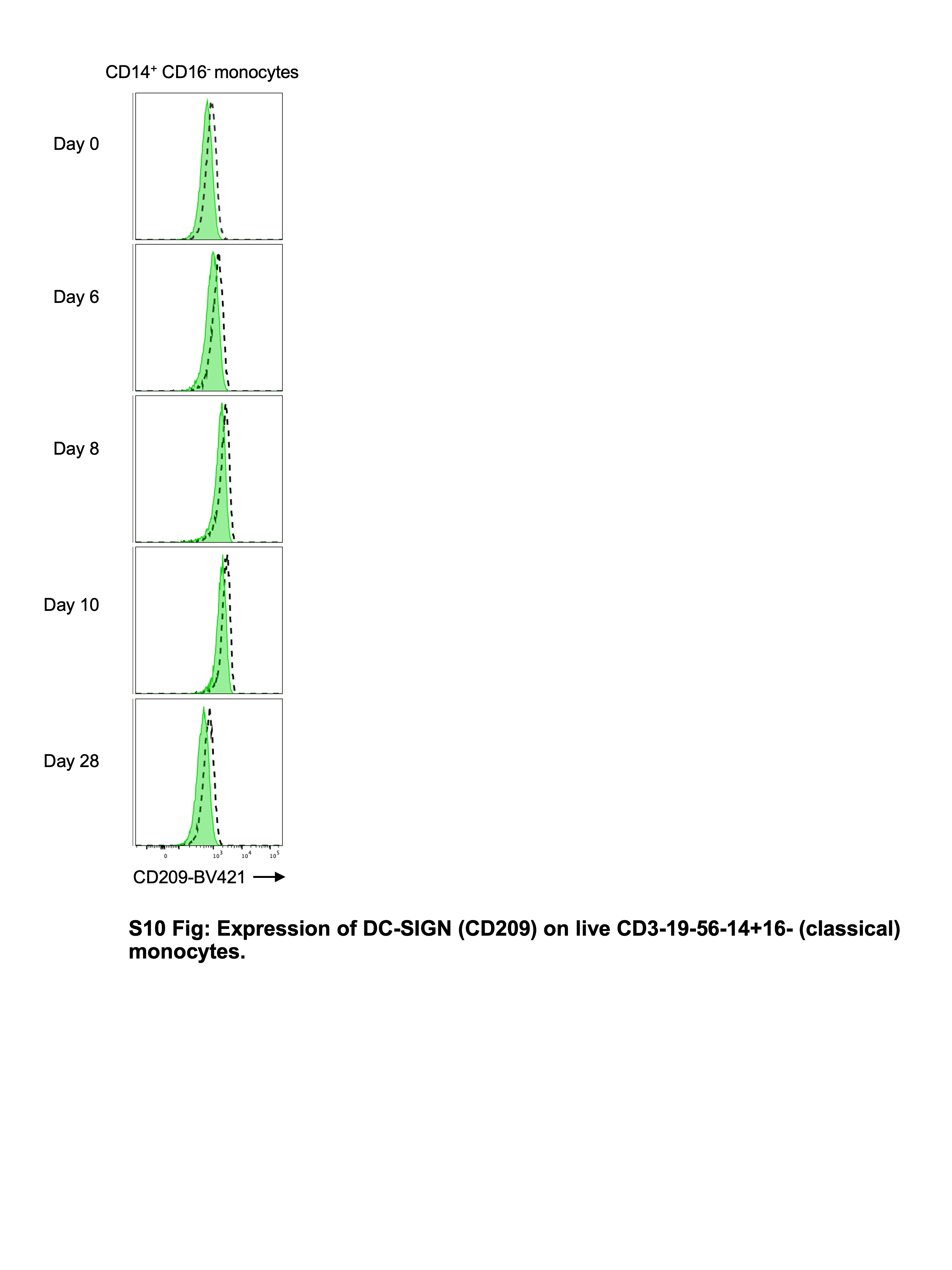

Supplement: S10 Fig — (TIFF) [file ppat.1011616.s010.tiff]

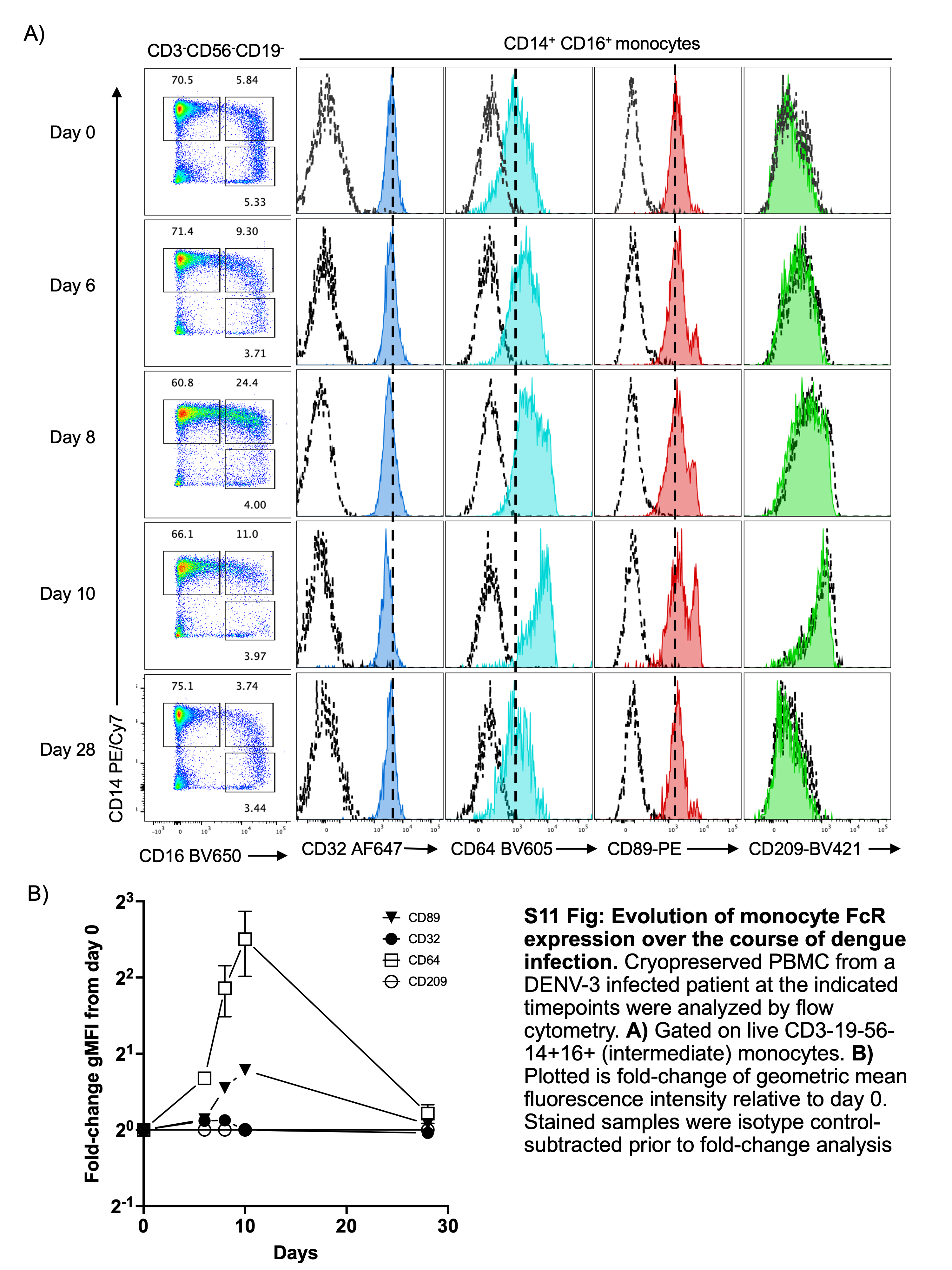

Supplement: S11 Fig — (TIFF) [file ppat.1011616.s011.tiff]

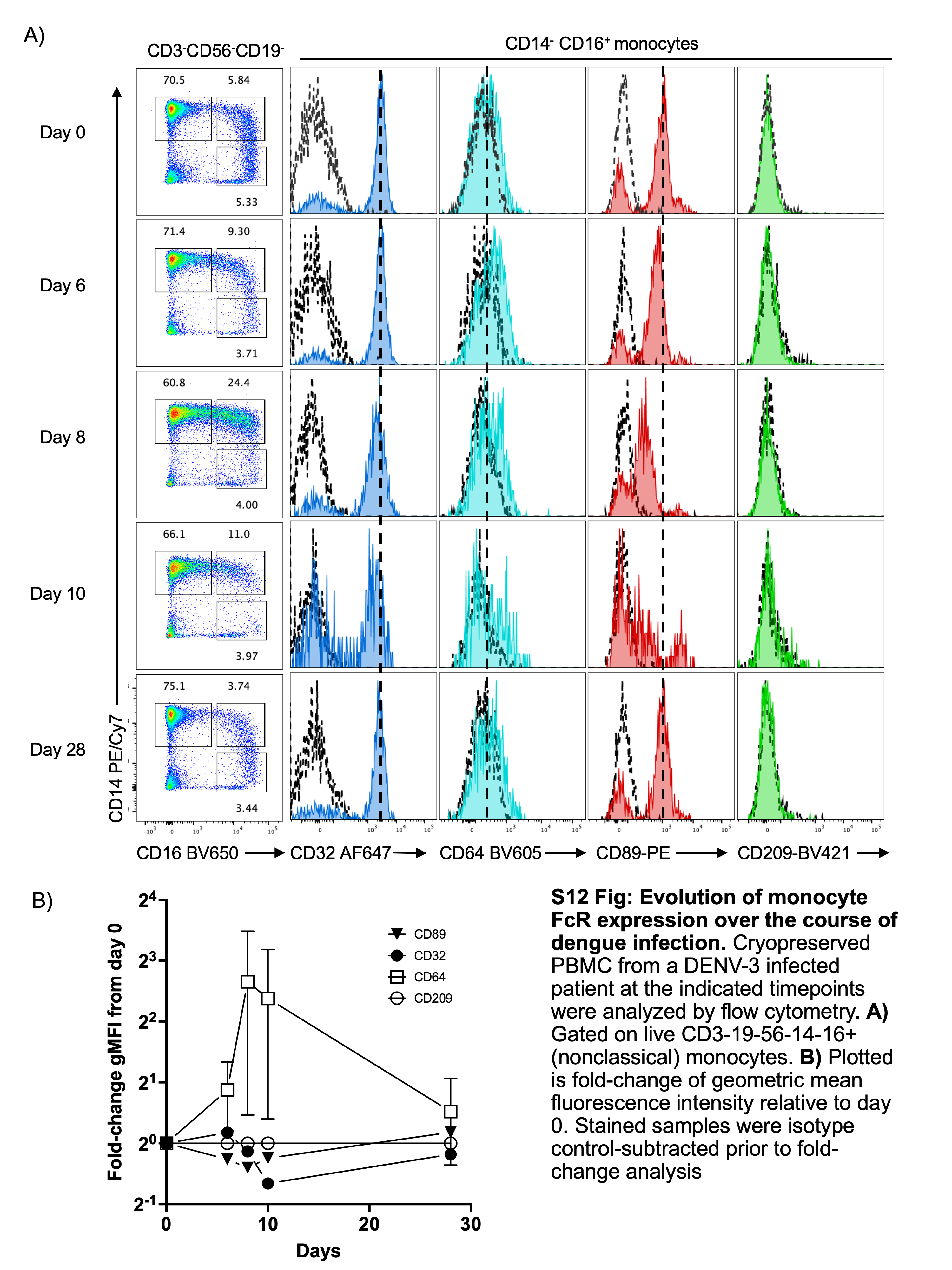

Supplement: S12 Fig — (TIFF) [file ppat.1011616.s012.tiff]

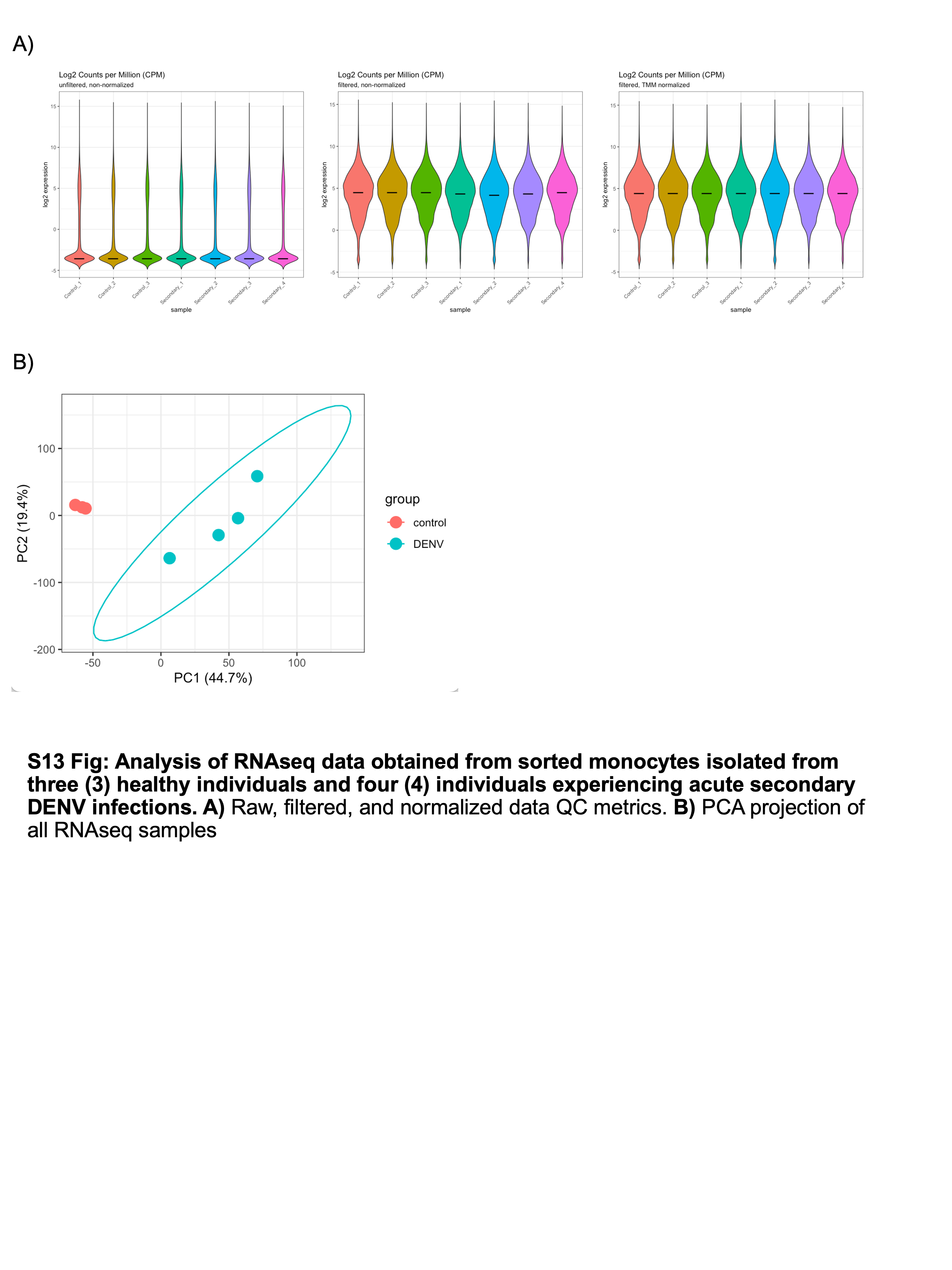

Supplement: S13 Fig — (TIFF) [file ppat.1011616.s013.tiff]
