# Supplementary material for: DENV-specific IgA contributes protective and non-pathologic function during antibody-dependent enhancement of DENV infection
Source: PLoS Pathog. 2023 Aug 28;19(8):e1011616. doi: 10.1371/journal.ppat.1011616 (PMC10491401; doi:10.1371/journal.ppat.1011616)
Supplement: S1 Table — (DOCX) [file ppat.1011616.s014.docx]

**S1 Table.** Antibodies used for flow cytometry

| **Antibody** | **Clone** | **Manufacturer** | **Catalog #** | **Lot #** | **Dilution** |
| --- | --- | --- | --- | --- | --- |
| CD14 PE-Cy7 | 63D3 | Biolegend | 367111 | B371303 | 1:80 |
| CD89 PE | A59 | Biolegend | 354103 | B313937 | 1:160 |
| CD32 AF647 | FUN-2 | Biolegend | 30312 | B378968 | 1:160 |
| CD3 BV785 | OKT3 | Biolegend | 317329 | B360623 | 1:320 |
| CD19 BV785 | HIB19 | Biolegend | 302239 | B355937 | 1:40 |
| CD56 BV785 | 5.1H11 | Biolegend | 362549 | B350905 | 1:40 |
| CD16 BV650 | 3G8 | Biolegend | 302041 | B272555 | 1:320 |
| CD64 BV605 | 10.1 | Biolegend | 305033 | B326358 | 1:80 |
| CD209 BV421 | 9E9A8 | Biolegend | 330117 | B368886 | 1:40 |
| Live/Dead Aqua | N/A | Thermo | L34957 | 2204201 | 1:500 |
