# Supplementary material for: DENV-specific IgA contributes protective and non-pathologic function during antibody-dependent enhancement of DENV infection
Source: PLoS Pathog. 2023 Aug 28;19(8):e1011616. doi: 10.1371/journal.ppat.1011616 (PMC10491401; doi:10.1371/journal.ppat.1011616)
Supplement: S2 Table — (DOCX) [file ppat.1011616.s015.docx]

**S2 Table.** Samples information for RNAseq analysis

| **Sample** | **Disease state** | **Primary/ Secondary** | **Days post symptom onset** | **SRA accession numbers** |
| --- | --- | --- | --- | --- |
| Control #1 | Healthy | -- | -- | SRR14714815, SRR14714816 |
| Control #2 | Healthy | -- | -- | SRR14714817, SRR14714818 |
| Control #3 | Healthy | -- | -- | SRR14714819, SRR14714820 |
| DENV #1 | Dengue | Secondary | 3 | SRR14714821, SRR14714822 |
| DENV #2 | Dengue | Secondary | 3 | SRR14714825, SRR14714826 |
| DENV #3 | Dengue | Secondary | 3 | SRR14714829, SRR14714830 |
| DENV #4 | Dengue | Secondary | 6 | SRR14714831, SRR14714832 |
