# Supplementary material for: DENV-specific IgA contributes protective and non-pathologic function during antibody-dependent enhancement of DENV infection
Source: PLoS Pathog. 2023 Aug 28;19(8):e1011616. doi: 10.1371/journal.ppat.1011616 (PMC10491401; doi:10.1371/journal.ppat.1011616)
Supplement: S3 Table — (DOCX) [file ppat.1011616.s016.docx]

**S3 Table.** Differential gene expression analysis of classical monocytes isolated from health donors and donors experiencing a secondary DENV infection.

| **geneID** | **logFC** | **AveExpr** | **t** | **P.Value** | **adj.P.Val** | **B** |
| --- | --- | --- | --- | --- | --- | --- |
| IFI27 | 10.2489945 | 7.55891631 | 14.5889384 | 1.88E-07 | 0.00205314 | 5.02792375 |
| SIGLEC1 | 4.08169594 | 8.02538962 | 13.5768754 | 3.44E-07 | 0.00205314 | 6.96311845 |
| MT-CO1 | -2.6933425 | 11.1319056 | -11.639306 | 1.25E-06 | 0.0029717 | 5.96186251 |
| COX7B | 2.44341194 | 4.57699837 | 9.31962986 | 7.66E-06 | 0.0083611 | 3.86197832 |
| LGALS3BP | 3.68250109 | 8.51542444 | 8.38090067 | 1.79E-05 | 0.00868526 | 3.48772847 |
| CMPK2 | 3.11863423 | 7.36060956 | 8.27866238 | 1.97E-05 | 0.00868526 | 3.38790804 |
| LY6E | 2.61114444 | 8.44386216 | 8.30123698 | 1.92E-05 | 0.00868526 | 3.41680499 |
| DDIT4 | 2.35844209 | 7.80447355 | 8.4165169 | 1.73E-05 | 0.00868526 | 3.51999557 |
| IFI44L | 3.88365149 | 6.61481096 | 8.13405878 | 2.26E-05 | 0.0089647 | 3.21630968 |
| C2 | 2.83117686 | 6.52669291 | 8.10589223 | 2.32E-05 | 0.0089647 | 3.21570487 |
| RNASE2 | 3.2314406 | 8.51350335 | 7.95116085 | 2.70E-05 | 0.00946248 | 3.08932127 |
| IFI44 | 2.66377249 | 6.62603271 | 7.92046844 | 2.78E-05 | 0.00947356 | 3.04962898 |
| CXCL8 | 4.37715476 | 7.80563697 | 7.36923759 | 4.86E-05 | 0.01123791 | 2.51936202 |
| IFITM3 | 3.87517282 | 9.99605564 | 7.35230721 | 4.94E-05 | 0.01123791 | 2.49409949 |
| CXCR2P1 | 3.16995369 | 7.27503266 | 7.20832203 | 5.75E-05 | 0.01123791 | 2.35537814 |
| EPSTI1 | 2.91152652 | 6.70963051 | 7.31391612 | 5.14E-05 | 0.01123791 | 2.46282197 |
| S100A8 | 2.42632168 | 11.9889936 | 7.32045556 | 5.11E-05 | 0.01123791 | 2.46117957 |
| USMG5 | 2.06497814 | 4.69443708 | 7.16747118 | 6.00E-05 | 0.01131988 | 2.23018716 |
| CD79B | 2.07503639 | 3.55664639 | 7.12404978 | 6.29E-05 | 0.01153348 | 1.90884465 |
| NECTIN2 | 2.31455039 | 5.2462809 | 7.10456216 | 6.42E-05 | 0.01159748 | 2.2163691 |
| PLAC8 | 3.94679542 | 7.98714436 | 7.00800152 | 7.12E-05 | 0.01212949 | 2.14369136 |
| CLU | 5.56208044 | 6.20759734 | 6.96204161 | 7.48E-05 | 0.01242183 | 2.01324741 |
| IFITM1 | 6.16134309 | 7.65589728 | 6.89698075 | 8.03E-05 | 0.01243401 | 2.02569553 |
| TYMS | 6.94068232 | -1.852099 | 6.76420554 | 9.30E-05 | 0.0128281 | -2.7495712 |
| OASL | 3.40929345 | 7.03442864 | 6.74400474 | 9.51E-05 | 0.0128281 | 1.86849387 |
| FAM69A | 2.42992763 | 2.31295155 | 6.74489569 | 9.50E-05 | 0.0128281 | 0.89476905 |
| CDKN1A | 2.12105383 | 9.22988613 | 6.73752483 | 9.57E-05 | 0.0128281 | 1.83504626 |
| CKS2 | 2.21936954 | 2.07320613 | 6.60116984 | 0.00011151 | 0.01385068 | 0.65011191 |
| MX1 | 2.50212032 | 9.34522517 | 6.53964307 | 0.00011954 | 0.01397452 | 1.61313751 |
| IFI6 | 3.3814124 | 8.57094734 | 6.34988522 | 0.00014856 | 0.01570248 | 1.40339616 |
| TPPP3 | -2.5765284 | 3.83052086 | -6.3743169 | 0.00014442 | 0.01570248 | 1.34110323 |
| HSPA1A | -2.0838389 | 7.5845034 | -6.1945003 | 0.00017808 | 0.01616251 | 1.22315157 |
| TCN2 | 2.57952429 | 7.49625287 | 6.15663641 | 0.00018621 | 0.01626092 | 1.18651646 |
| USP18 | 4.54257296 | 5.30054292 | 6.12178962 | 0.00019405 | 0.01664639 | 1.11356496 |
| IFIT3 | 3.48482969 | 7.76647354 | 6.00968474 | 0.00022181 | 0.01685759 | 1.01618229 |
| SMIM4 | 2.1036261 | 2.2928707 | 6.04861886 | 0.00021171 | 0.01685759 | 0.4532808 |
| PLCB1 | -2.1652975 | 4.03389742 | -5.9552906 | 0.00023682 | 0.01707436 | 0.9567221 |
| RTN1 | -3.4852722 | 5.84838579 | -5.9219247 | 0.00024657 | 0.01715926 | 0.95650971 |
| GMPR | 3.20335524 | 4.65003674 | 5.88430449 | 0.00025809 | 0.0172733 | 0.86969807 |
| TRIP6 | 2.02913347 | 3.07436292 | 5.79137808 | 0.00028915 | 0.01777226 | 0.57035258 |
| P2RY6 | 3.8856636 | 4.0153183 | 5.76570948 | 0.00029843 | 0.01788172 | 0.57342117 |
| SPAG17 | -5.3806978 | -2.7640061 | -5.7591066 | 0.00030087 | 0.01793772 | -2.6367978 |
| LAMB2 | -2.7971873 | 3.31840314 | -5.6933902 | 0.00032636 | 0.01879956 | 0.54905241 |
| ETV7 | 3.75567195 | 4.45839679 | 5.68629674 | 0.00032925 | 0.01887479 | 0.59939736 |
| EMP1 | 2.80747826 | 4.06656671 | 5.67210639 | 0.00033511 | 0.01895579 | 0.60045713 |
| ITGB7 | 2.26927612 | 7.5769034 | 5.61659948 | 0.00035916 | 0.01944426 | 0.52128035 |
| CCL3 | 4.64870869 | 7.02074106 | 5.59097249 | 0.00037089 | 0.01972418 | 0.55311925 |
| HIST2H2AA4 | 2.15127898 | 6.82791693 | 5.46262657 | 0.00043626 | 0.02114623 | 0.34661886 |
| HERC2P9 | -2.1728692 | 3.07505835 | -5.4129797 | 0.00046482 | 0.02186191 | 0.2415528 |
| APOBEC3A | 2.14998571 | 8.5557911 | 5.3929438 | 0.00047691 | 0.02210741 | 0.21784498 |
| LAG3 | 6.35353004 | -2.1786443 | 5.23940801 | 0.00058174 | 0.02319851 | -3.0378367 |
| CTSL | 2.56798149 | 6.24124976 | 5.25070998 | 0.00057322 | 0.02319851 | 0.10796577 |
| BCL2A1 | 2.49998051 | 7.14769726 | 5.2035483 | 0.00060966 | 0.02319851 | 0.0036396 |
| HERC2P3 | -2.1377191 | 2.78004869 | -5.2265036 | 0.00059162 | 0.02319851 | -0.0228163 |
| PID1 | -2.3598692 | 5.2039148 | -5.1953963 | 0.00061621 | 0.02319851 | 0.07554157 |
| VAMP5 | 2.85588831 | 6.59649306 | 5.17243139 | 0.00063507 | 0.02337208 | -0.0048788 |
| RP11-1212A22.1 | -2.3855169 | 3.02316016 | -5.1509752 | 0.00065326 | 0.02353297 | -0.0614675 |
| IFIT1 | 3.35708351 | 5.98234247 | 5.14530569 | 0.00065815 | 0.02357102 | 0.00663679 |
| COLEC12 | -2.7786293 | 1.06625758 | -5.1346581 | 0.00066746 | 0.02375771 | -1.0185485 |
| SPOCK3 | -4.9535677 | -2.4714168 | -5.1027375 | 0.00069623 | 0.02423966 | -2.7936064 |
| OAS3 | 2.31587421 | 9.05387551 | 4.98301671 | 0.0008167 | 0.02642864 | -0.3334256 |
| RNASE3 | 2.70284446 | 3.95118314 | 4.96854388 | 0.00083272 | 0.02683625 | -0.2069679 |
| TRBC2 | 6.11747339 | -1.827965 | 4.94164335 | 0.00086342 | 0.0274142 | -3.086966 |
| MT1E | 4.25699606 | 0.21756822 | 4.93515882 | 0.000871 | 0.0274142 | -2.3934917 |
| SEZ6L | -2.7223227 | 1.46275631 | -4.9140224 | 0.00089621 | 0.02752701 | -0.9494304 |
| TRAF3IP2 | 2.17104696 | 3.27091263 | 4.89778471 | 0.00091612 | 0.02755615 | -0.3408662 |
| C1orf54 | 2.01611276 | 2.71648409 | 4.89864149 | 0.00091505 | 0.02755615 | -0.4315697 |
| ADAMTS5 | -5.844772 | -1.4418959 | -4.9008816 | 0.00091228 | 0.02755615 | -2.7379722 |
| PALLD | -2.372897 | 3.27987 | -4.8153723 | 0.00102476 | 0.02902836 | -0.4107617 |
| PRF1 | 7.61848326 | -0.8022325 | 4.75253884 | 0.00111692 | 0.02916433 | -3.0662759 |
| RAB3IL1 | 5.93310991 | -1.9473014 | 4.74408372 | 0.00112998 | 0.02916433 | -3.145523 |
| CXCL2 | 5.83373739 | 4.57783145 | 4.76116685 | 0.00110375 | 0.02916433 | -0.5848545 |
| FKBP11 | 2.11868889 | 2.34009484 | 4.76009093 | 0.00110538 | 0.02916433 | -0.6937578 |
| ACSM3 | -3.261384 | 0.26648413 | -4.7522083 | 0.00111742 | 0.02916433 | -1.8640703 |
| RSAD2 | 3.43805058 | 6.51800087 | 4.66738116 | 0.00125633 | 0.03032488 | -0.6612825 |
| ZBP1 | 2.73009 | 4.35955024 | 4.66158906 | 0.00126647 | 0.03044637 | -0.5727418 |
| FLT3 | 2.23452164 | 4.19661505 | 4.65400721 | 0.00127988 | 0.0306451 | -0.5827116 |
| SERPING1 | 3.83746358 | 7.35654381 | 4.6261614 | 0.00133044 | 0.03122869 | -0.7612567 |
| SEL1L3 | 2.85771449 | 4.32306265 | 4.58568107 | 0.00140781 | 0.03191402 | -0.6702786 |
| CACNA2D3 | -2.9478162 | 2.66556113 | -4.5883861 | 0.0014025 | 0.03191402 | -0.7916453 |
| NET1 | 2.11792697 | 1.88246374 | 4.58049542 | 0.00141807 | 0.03196422 | -1.0413173 |
| FCGR3B | 2.36691487 | 1.21962491 | 4.56136034 | 0.00145662 | 0.0320555 | -1.4481199 |
| SULF2 | -2.210227 | 8.66922888 | -4.5627002 | 0.00145388 | 0.0320555 | -0.9185308 |
| TXNDC16 | -2.0205171 | 2.51735923 | -4.5368963 | 0.00150754 | 0.0326134 | -0.8272468 |
| FCER1A | -5.2329791 | 1.35749528 | -4.5358428 | 0.00150978 | 0.0326134 | -1.7963182 |
| IGHV1-69 | 8.59795897 | -0.8834566 | 4.51315639 | 0.00155879 | 0.03307306 | -3.1243201 |
| MS4A4A | 3.36572872 | 4.6784702 | 4.50805999 | 0.00157004 | 0.03314938 | -0.7728857 |
| TESPA1 | 4.41211735 | 1.41088853 | 4.48885755 | 0.00161319 | 0.03330597 | -1.8734804 |
| RNASE1 | 7.53938389 | 3.89401444 | 4.48143622 | 0.00163021 | 0.03345725 | -1.4986743 |
| MYBL2 | 8.67266648 | -0.9028686 | 4.44936729 | 0.001706 | 0.03375621 | -3.1429554 |
| CXCR3 | 7.02937027 | -1.3034679 | 4.42583912 | 0.001764 | 0.03427939 | -3.1740784 |
| AVPI1 | 2.08269743 | 3.79020609 | 4.42260914 | 0.00177213 | 0.03434085 | -0.8813826 |
| TDRD6 | -3.2403619 | 0.60568569 | -4.4129961 | 0.00179655 | 0.03451056 | -1.8647632 |
| CD38 | 2.75829133 | 5.27020045 | 4.40291409 | 0.00182255 | 0.03463772 | -0.9588762 |
| PPARG | 4.254236 | -0.4377972 | 4.39665185 | 0.00183891 | 0.03474979 | -2.9859276 |
| GGTA1P | -2.3661213 | 3.11999202 | -4.3826088 | 0.00187615 | 0.03523033 | -0.9488942 |
| QPCTL | -2.017083 | 1.15963663 | -4.3726248 | 0.00190313 | 0.03562462 | -1.4559217 |
| LGR4 | -5.2546072 | -0.4452635 | -4.3676071 | 0.00191684 | 0.03582507 | -2.8353251 |
| CD1C | -2.3368925 | 3.23226062 | -4.3574539 | 0.00194491 | 0.0361687 | -0.9732388 |
| ZNF219 | -2.230907 | 1.19294991 | -4.3441955 | 0.00198223 | 0.03647787 | -1.479141 |
| NCOA7 | 2.27180346 | 5.3183075 | 4.34112095 | 0.001991 | 0.03647998 | -1.0670456 |
| BLNK | 2.95105663 | 2.73921874 | 4.31739625 | 0.00206003 | 0.03699369 | -1.1568611 |
| IL32 | 5.02311489 | -0.2726235 | 4.29879683 | 0.00211594 | 0.03735109 | -3.0699314 |
| CYP27A1 | -2.3745228 | 6.93945985 | -4.2914191 | 0.00213857 | 0.03739379 | -1.2691364 |
| ZBTB46 | -2.8274057 | 0.06894691 | -4.2853536 | 0.00215736 | 0.03739379 | -2.1641562 |
| ELOVL3 | 2.54377564 | 2.69629427 | 4.26886154 | 0.00220937 | 0.03760882 | -1.1814611 |
| NFKBIZ | 2.14815447 | 9.12344043 | 4.22871907 | 0.00234163 | 0.03849117 | -1.4104935 |
| AL022067.1 | 5.99976867 | -0.2783115 | 4.22326646 | 0.00236024 | 0.03871182 | -3.2186414 |
| SAMD9L | 2.09931916 | 6.7394137 | 4.18471339 | 0.0024964 | 0.03942661 | -1.4101041 |
| KAZALD1 | 4.1403915 | -0.0499374 | 4.17565875 | 0.00252958 | 0.0397401 | -2.8492286 |
| GBP1 | 3.15365948 | 8.29387546 | 4.17681995 | 0.0025253 | 0.0397401 | -1.4667333 |
| CYBRD1 | -2.0974004 | 5.04019627 | -4.1761636 | 0.00252772 | 0.0397401 | -1.2992877 |
| KCND1 | -4.2473597 | -1.8401304 | -4.1696868 | 0.00255172 | 0.03974385 | -3.0557671 |
| LCK | 5.91972479 | -2.0359509 | 4.1578903 | 0.00259607 | 0.04009784 | -3.3105761 |
| BATF2 | 2.32897445 | 3.47226302 | 4.15821288 | 0.00259485 | 0.04009784 | -1.2309272 |
| PRR5 | 2.02985671 | 1.66862393 | 4.14945919 | 0.00262827 | 0.04034525 | -1.529677 |
| EPHB4 | -2.0910743 | 1.77195109 | -4.1398785 | 0.00266537 | 0.04045741 | -1.4532668 |
| MT2A | 2.79948489 | 6.19178598 | 4.09924999 | 0.00282905 | 0.04126061 | -1.4728624 |
| CDK5R1 | -2.0069458 | 1.78181052 | -4.1007426 | 0.00282285 | 0.04126061 | -1.4887255 |
| GLDN | 6.01314111 | -1.9495759 | 4.09362335 | 0.00285255 | 0.04142973 | -3.318788 |
| CADM1 | 6.43779174 | -0.1561232 | 4.07718467 | 0.0029224 | 0.04171802 | -3.2596111 |
| HIST1H1C | 2.77559636 | 3.45126889 | 4.0671061 | 0.00296613 | 0.04210493 | -1.3524599 |
| ACOX2 | 3.59316621 | 1.97320894 | 4.0297998 | 0.00313415 | 0.04297743 | -1.8081987 |
| IFIT2 | 2.24075125 | 7.46258782 | 4.00972432 | 0.00322873 | 0.04335515 | -1.7041505 |
| CSGALNACT1 | -2.7069056 | 2.97055347 | -3.999818 | 0.00327652 | 0.04356921 | -1.4477603 |
| TPRG1 | 2.21933707 | 1.40439991 | 3.99108449 | 0.00331927 | 0.04377441 | -1.7926627 |
| S100P | 3.97943452 | 1.84061999 | 3.9760874 | 0.00339407 | 0.04419737 | -2.0589157 |
| HEG1 | 2.72848895 | 3.98720221 | 3.97781211 | 0.00338538 | 0.04419737 | -1.4716163 |
| OPTN | 2.39862097 | 3.42267192 | 3.96563084 | 0.00344729 | 0.04443833 | -1.4824123 |
| HIST2H4A | 2.46641467 | 1.21044524 | 3.9613444 | 0.00346936 | 0.04448239 | -1.9592829 |
| NRP2 | -2.5739541 | 1.32435505 | -3.9112019 | 0.00373898 | 0.04663552 | -1.8681983 |
| CCL3L3 | 3.18216098 | 5.71825959 | 3.88972455 | 0.00386119 | 0.04739612 | -1.7158301 |
| H1F0 | 2.6042655 | 5.99063085 | 3.89123691 | 0.00385245 | 0.04739612 | -1.7685635 |
| RP11-367G6.3 | 2.31685268 | 0.82736128 | 3.87905667 | 0.00392346 | 0.04783572 | -2.1620711 |
| TPST1 | 5.21163745 | 1.35001895 | 3.86519407 | 0.00400598 | 0.04784201 | -2.5284595 |
| TNNT1 | 5.17792491 | 3.49688965 | 3.86481909 | 0.00400823 | 0.04784201 | -1.729091 |
| NKG7 | 3.51122693 | 4.83382316 | 3.86882044 | 0.00398421 | 0.04784201 | -1.6476313 |
| IL10 | 2.29803426 | 1.21638328 | 3.86766644 | 0.00399113 | 0.04784201 | -2.0415473 |
| LDLR | 2.29015313 | 6.65656421 | 3.86802583 | 0.00398897 | 0.04784201 | -1.8742143 |
| STARD4 | 2.05778141 | 3.85471449 | 3.83912989 | 0.00416614 | 0.04918262 | -1.6683773 |
| F8A3 | 5.10857855 | 3.51599396 | 3.83318774 | 0.0042036 | 0.04928587 | -1.7008201 |
| FCGR2C | -2.4181697 | 3.05813549 | -3.8338894 | 0.00419916 | 0.04928587 | -1.6578263 |
| DUSP2 | 2.31544366 | 8.81665456 | 3.81772405 | 0.00430276 | 0.04987691 | -2.0265022 |
