# Supplementary material for: DENV-specific IgA contributes protective and non-pathologic function during antibody-dependent enhancement of DENV infection
Source: PLoS Pathog. 2023 Aug 28;19(8):e1011616. doi: 10.1371/journal.ppat.1011616 (PMC10491401; doi:10.1371/journal.ppat.1011616)
Supplement: Attachment — Submitted filename: Response to reviewers.pdf [file ppat.1011616.s018.pdf]

Reviewer #1:

My only major concern is the data in Figure 5. ADE is not known to occur during primary infection and the changes in the expression of these receptors are occurring at a time when neither IgA nor IgG antibodies are expressed at high or even detectable levels. While it can be argued that a similar trend in the expression of these Fc receptors could be expected in secondary dengue, such an argument would ignore the known significant alterations in host response during ADE that can be detected as early as 24 hours from infection (Chan et al, mSphere 2019; 4:e00528-19). I do not think that omitting this set of data from the manuscript weakens the evidence that monomeric IgA antibodies are unlikely players in antibody-enhanced dengue and would recommend that the authors remove this data for a more focused paper.

We thank the reviewer for this suggestion. We wish to emphasize that our objective with the analysis presented in Figure 5 was not to identify and quantify transcriptional signatures of ADE in monocytes during DENV infection (as was elegantly demonstrated by Chan et al) but to determine if DENV-elicited inflammation modulated the expression of FcαR. FcγR expression on macrophages and monocytes is known to change in response to cytokine stimulation, TLR engagement, or viral infection. However, we failed to identify any previously-published report assessing FcαR expression on these cells during an acute viral infection. Given that we are postulating that IgA is unable to facilitate ADE due to both the lower affinity of IgA for FcαR and the lower expression of FcαR relative to FcγR we felt it was important to assess FcαR expression on monocytes during an acute DENV infection.

However, we recognize that the kinetics and composition of DENV-elicited inflammation are different in primary and secondary DENV infections, and therefore we should not solely rely on the data generated by our experimental primary infections to address this concern. Therefore, we have included additional data in this resubmission (Figure 5C, Figure 5D, Supplemental Figure 13, and Supplemental Table 3) quantifying the expression of FcαR in monocytes isolated from healthy individuals and individuals experiencing acute secondary DENV infections. The re-analysis of a publicly available dataset (NCBI GEO accession GSE176079) revealed that FcαR expression in conventional monocytes was not increased during secondary DENV infection, mirroring what was observed at a protein level during a primary DENV infection.

We have additionally added language to the discussion section of our revised submission better placing this observation in context with when virus and antibodies are thought to co-circulate during both primary and secondary dengue and the potential relationship to disease severity.

Reviewer #2:

1. Fig 1 and 2: it is not clear how "infection rate" of U937 or monocyte-derived macrophages is measured (Fig 1B and 2C). Is this calculated based on the staining of DENV-infected cells with an anti-prM PE antibody as shown in Supplementary Figure 2 A-B (U937 showing approx. 10% infection) and Supplementary Fig 4 A-B (monocyte-derived macrophages showing 17% infection)? These anti prM ab staining profiles are not very convincing. Could the authors show staining using a different anti DENV ab? Can the authors show DENV virions released by the infected cell to prove that DENV is replicating as well as entering the cell?

We have included additional data in Supplemental Figure 3 quantifying DENV infection in the U937 ADE assay using the anti-E antibody clone 4G2.

2. Figure 5 shows that FCgRI is highly expressed in dengue infected individuals while FCgRII is not. Can the authors demonstrate in the experiments shown in Fig 1B and 1C that FcgRI is indeed playing a role in ADE and the effect is not only mediated by FcgRII (for example by using blocking abs to FcgRI/IIa)? This data would strengthen the validity of the in vitro data for the clinical context. Could the authors please comment on the timing of upregulation of FcgRI in patient monocytes, would this fit with the timing of when ADE is thought to occur during dengue infection?

We wish to thank the reviewer for this interesting suggestion. We have added additional data to our study as a new Supplement Figure 4 where we spiked FcγRIIa and FcγRI blocking antibodies into our U937 ADE assay. We observed that both FcgRIIa blocking antibodies (clone IV.3) and FcgRI blocking antibodies (clone 10.1) antagonized IgG-

mediated ADE in this system, with the addition of both antibodies nearly ablating IgG-mediated infection. This matches several previously published results using similar infection systems.

However, we wish to specifically address the reviewer comment that "*FcgRI is highly expressed in dengue infected individuals while FCγRII is not***". While FcγRI expression is indeed highly expressed on monocytes during acute DENV infection, FcγRIIa expression is also abundant. The old Figure 5B depicted the relative change in receptor expression relative to pre-infection levels, not the absolute expression of the receptor. We wish to apologize for the previously unclear presentation of the data and have moved the representative flow plots of FcγRI, FcγRIIa, and FcαR staining from supplemental data to the primary figure as suggested below.**

Minor Issues: Editorial and Data Presentation Modifications

Reviewer #1:

Lines 129-130: K562 is widely used but is not the gold standard for ADE assays. Primary monocytes, monocyte-derived macrophages/dendritic cells would make a more convincing gold standard.

We agree with the reviewer and have modified the language in our manuscript accordingly.

Line 142 and elsewhere: Please specify the MOI used for all the experiments.

We apologize for omitting this key detail in our initial submission All DENV stocks in this study were used at a dilution shown to achieve a 20% infection rate in DC-SIGN expressing U937 cells. This corresponds to an MOI of 3 when these same viral stocks were titrated on Vero cells.

Figure 3: It would be useful to show the concentrations of these cytokines for each of the experimental conditions in the supplementary data. Such data would complement the fold changes shown in this Figure.

These data have been added to our revised submission as Supplemental Figure 8.

Figure 4c. Why is there a positive RT-qPCR finding for the no-DENV control? Perhaps the authors would consider showing the detection limit of the RT-qPCR used in this experiment?

We thank the reviewer for noting this signal. There was a significant amount of cellular RNA (U937 cells and macrophage) in the samples analyzed by qRT-PCR assay due to the nature of the virus-binding assay. The presence of this cellular RNA resulted in a small background PCR signal even in those samples that were not incubated with DENV. As the cellular RNA content is the same across all conditions this does not impact the interpretation of the data.

Reviewer #2:

1. In Fig 5 it would be useful to show some flow cytometry plots for a representative donor to show the monocyte populations that the authors are looking at and how the markers of interest are expressed by classical monocytes for B. The authors could move this data now in supplem figs to the main figure.

We have moved these data from supplementary material to Figures 5A in our revised submission as suggested.

2. While I agree that in vitro monocyte-derived macrophages are a good model for ex vivo macrophages, and I

appreciate the difficult of working with ex vivo primary macrophages, in my opinion it is not correct to define monocyte-derived macrophages as “primary” cells as they are generated in vitro.

We agree with the reviewer and have modified the language in our manuscript accordingly.
